# *Parahenipavirus langyaense* (Langya virus): Spillover ecology, noncanonical receptor-mediated entry, and future control strategies

**DOI:** 10.1016/j.onehlt.2026.101521

**Published:** 2026-07-16

**Authors:** Liang Shen, Yanfei Tong, Wenqi Chai, Jiali Sun, Jianzhong Zhao, Lijuan Yin, Yang Yang, Chunhua Wang, Wenjie Tan, Ji Zhang

**Affiliations:** a Department of Central Laboratory, Xiangyang Central Hospital, Affiliated Hospital of Hubei University of Arts and Science, Hubei Province, Xiangyang 441021, China; b Department of Neurology, Xiangyang Central Hospital, Affiliated Hospital of Hubei University of Arts and Science, Hubei Province, Xiangyang 441021, China; c Department of Clinical Laboratory, Xiangyang No. 1 People's Hospital, Hubei University of Medicine, Xiangyang 441000, Hubei Province, China; d State Key Laboratory of Food Nutrition and Safety, Key Laboratory of Industrial Microbiology, Ministry of Education, Tianjin Key Laboratory of Industry Microbiology, National and Local United Engineering Lab of Metabolic Control Fermentation Technology, China International Science and Technology Cooperation Base of Food Nutrition/Safety and Medicinal Chemistry, College of Biotechnology, Tianjin University of Science & Technology, Tianjin 300457, China; e Shenzhen Key Laboratory of Pathogen and Immunity, National Clinical Research Center for infectious disease, State Key Discipline of Infectious Disease, Shenzhen Third People's Hospital, Second Hospital Affiliated to Southern University of Science and Technology, Shenzhen, China; f Department of Clinical Laboratory, Xiangyang Central Hospital, Affiliated Hospital of Hubei University of Arts and Science, Hubei Province, Xiangyang 441021, China; g NHC Key Laboratory of Biosafety, National Institute for Viral Disease Control and Prevention, Chinese Center for Disease Control and Prevention, Beijing 102206, China

**Keywords:** Langya virus, *Parahenipavirus*, Shrews, Noncanonical receptor, Structure-guided countermeasure strategies

## Abstract

Langya virus (LayV), classified in the species *Parahenipavirus langyaense*, is a newly identified zoonotic paramyxovirus discovered in febrile patients in eastern China in recent years. The discovery of LayV has expanded the field beyond the prevailing paradigm of highly pathogenic, bat-borne henipaviruses represented by Nipah virus (NiV) and Hendra virus (HeV), toward a *parahenipavirus* spillover model shaped by shrews, rodents, and agricultural ecological interfaces. Reported cases have mainly presented with fever, fatigue, cough, and vomiting, and may be accompanied by leukopenia, thrombocytopenia, and abnormalities in hepatic and renal function. However, owing to the limited number of cases, restricted surveillance coverage, incomplete follow-up data, and the lack of standardized serological tools, the true burden of infection, full spectrum of natural hosts, role of intermediate hosts, and animal-to-human transmission pathways remain unresolved. Sustained human-to-human transmission has not been confirmed. In recent years, accumulating evidence from viromic analyses of shrew lung tissues along the eastern coast of China, positive detection in the Ussuri white-toothed shrew (*Crocidura lasiura*) in South Korea, characterization of the LayV genome architecture, P-gene RNA editing, ELISA/multiplex RT-qPCR/CRISPR-Cas12a diagnostic platforms, and structure-based F/G antigen engineering and antibody development has begun to move LayV research from pathogen discovery toward mechanistic dissection and the construction of countermeasure platforms. In this Review, we systematically summarize the discovery of LayV, clinical features and epidemiological evidence, taxonomy and molecular evolution, animal host ecology, noncanonical receptor-mediated entry mechanisms, diagnostic approaches, and immunological intervention strategies. We further propose key research priorities centered on One Health surveillance, identification of the unknown receptor, structure-guided F/G antigen design, and integrated sampling across animal, environmental, and human populations.

## 1 Introduction

Pandemics and regional outbreaks of viral infectious diseases have repeatedly demonstrated that emerging pathogens can not only precipitate severe public health crises, but also exert profound effects on economic activity, social governance, and the resilience of health systems. Systematic investigation of emerging viruses is essential for defining their origins, transmission dynamics, and mechanisms of pathogenesis, thereby providing a scientific foundation for early warning, risk assessment, and the development of prevention and control systems. For decades, research on henipaviruses has focused predominantly on NiV and HeV. These viruses can cause severe respiratory and neurological disease and form complex transmission chains linking fruit bats, wild mammals, livestock, and humans, making them among the most prominent emerging zoonotic pathogens within the family *Paramyxoviridae* [1], [2], [3]. However, the One Health framework underscores the deep interconnection among humans, domestic animals, wildlife, and their socioecological environments. Interpreting emerging viruses solely through the lens of a single host species or an isolated clinical outbreak risks underestimating the contributions of agricultural ecosystems, small wild mammals, and environmental exposures to cross-species spillover [4].

Langya virus (LayV) was first identified through metagenomic sequencing during sentinel surveillance of febrile patients with histories of animal exposure in Shandong, Henan, and neighboring regions of China. Comparative genomic analysis indicated that LayV is relatively closely related to Mojiang virus (MojV), which was discovered in Yunnan in 2012. The original report described a virus detected in febrile patients that was phylogenetically distinct from previously known henipaviruses. A total of 35 acute LayV infections were reported, including 26 cases in which no other pathogen was detected, suggesting that the virus may be associated with a subset of febrile illnesses [5]. The significance of this discovery lies not only in the identification of a novel paramyxovirus capable of infecting humans, but also in the integration of clinical surveillance for fever of unknown origin, epidemiological clues from animal exposure, screening of small wild mammals, and viral genomic characterization into a relatively coherent chain of spillover recognition.

Although LayV has not yet been shown to pose a confirmed threat of human-to-human transmission, it provides an important model for understanding cross-species spillover of paramyxoviruses associated with non-bat hosts and for building forward-looking paramyxovirus preparedness platforms. Its scientific importance lies within a critical window characterized by limited case numbers, substantial uncertainty, and exceptional mechanistic relevance. Early commentaries generally framed LayV within the post-COVID-19 context of emerging pathogen surveillance, emphasizing that it represents a zoonotic risk signal requiring sustained monitoring, but not undue alarm [6], [7], [8]. More recent reviews have further noted that the clinical, molecular, and public health evidence for LayV is still accumulating. Current data are sufficient to support systematic investigation of LayV as an emerging zoonotic disease model, but remain insufficient to infer that it has acquired stable human-to-human transmissibility or pandemic potential [9].

Accordingly, this Review does not treat LayV as an isolated emerging-virus event. Instead, we position it as a representative model of shrew-associated *parahenipavirus* spillover and organize our discussion around three central questions: how the host ecology of LayV is structured, how the virus enters host cells, and whether diagnostic, antigen-engineering, and structure-guided countermeasure platforms should be developed in advance. Through this framework, we aim to provide a reference for elucidating the mechanisms of LayV infection, assessing the risk of cross-species transmission, and developing potential preventive and therapeutic strategies.

## 2 Pathogen discovery, clinical features, and epidemiological evidence

### 2.1 Febrile sentinel surveillance and the first identification of LayV

The discovery of LayV originated from sentinel surveillance of febrile patients in eastern China, rather than from a conventional investigation of a large-scale outbreak. The index cases had fever and a history of animal exposure; viral sequences were detected in clinical specimens, including throat swabs, and the genome was subsequently characterized. This discovery underscores the value of integrating clinical cohorts with fever of unknown origin, metagenomic sequencing, and targeted nucleic acid screening to identify low-frequency zoonotic spillover events before they develop into recognizable outbreaks. This mode of discovery carries important implications for emerging-virus surveillance: many zoonotic viruses may not first come to public-health attention as clustered outbreaks, but instead may initially present as sporadic, nonspecific febrile or respiratory illness.

Unlike NiV, which has generated animal-to-human or human-to-human transmission chains in Malaysia, Bangladesh, India, and other settings, publicly reported LayV cases have largely been sporadic, with no clear epidemiological links among cases. On this basis, early public-health commentaries argued against equating LayV directly with the risk of “the next pandemic” and instead proposed that it should be regarded as a zoonotic spillover signal warranting sustained surveillance [10], [11]. This cautious framing sets the tone for subsequent interpretation: LayV should neither be sensationalized nor dismissed simply because the currently recognized case count remains small.

### 2.2 Clinical spectrum and levels of evidence

**Fig. 1:**
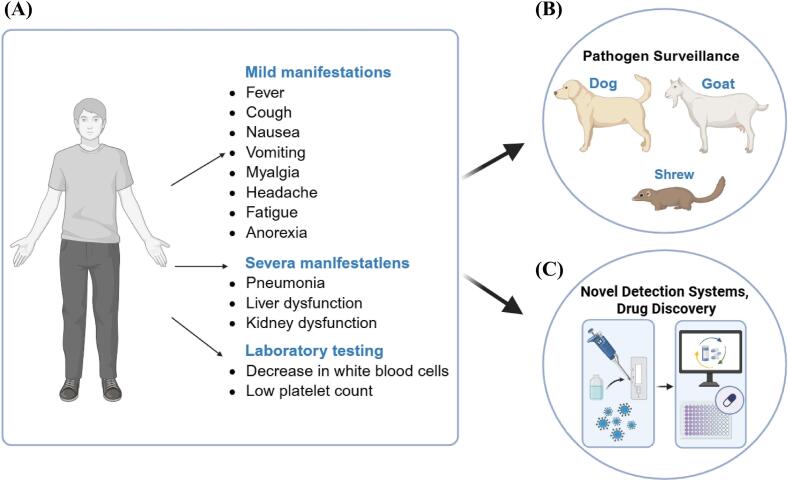
Clinical manifestations and control strategies for Langya virus (LayV) infection. (A) Clinical symptoms and laboratory features observed in patients with LayV infection. (B) Zoonotic pathogens that should be incorporated into febrile sentinel surveillance. (C) Development of next-generation diagnostic systems and antiviral therapeutics.

Reported LayV infections to date have been dominated by nonspecific systemic and respiratory manifestations. The original case series showed that, among patients in whom only LayV was detected, fever was the most consistent feature, followed by fatigue in approximately 54%, cough in approximately 50%, anorexia in approximately 50%, myalgia in approximately 46%, nausea in approximately 38%, and headache and vomiting in approximately 35% each. Laboratory abnormalities mainly included leukopenia, thrombocytopenia, and evidence of hepatic and renal dysfunction, suggesting that LayV infection may involve the respiratory system, hematologic compartment, and liver and kidney function **(**Fig. 1A**).** Viral loads were higher in patients with pneumonia than in those without pneumonia, indicating that the level of viral replication may be associated with certain clinical phenotypes; however, the available sample size remains insufficient to establish a robust model for predicting severe disease [5].

Interpretation of the clinical evidence requires careful distinction among primary studies, integrative reviews, and brief communications. To date, reported cases have not included definitive central nervous system involvement or fatal outcomes, and the overall clinical course appears milder than that of NiV or HeV infection. Nevertheless, because the number of cases is limited, follow-up remains incomplete, and the relationship between viral load and organ injury has not been systematically modeled, these observations should not be used to infer that LayV is intrinsically weakly pathogenic. Short reports describing thrombocytopenia, bleeding manifestations, or gingival bleeding may serve as clinical signals, but they should not be treated as the principal basis for assessing disease severity [12], [13], [14], [15]. A more rigorous formulation is that LayV can cause febrile illness accompanied by multisystem laboratory abnormalities, while its full clinical spectrum remains to be defined through prospective cohorts, standardized specimen collection, and longitudinal follow-up.

From the perspective of clinical management, no LayV-specific therapy is currently available. In the absence of approved antiviral agents or preventive vaccines, suspected or confirmed cases primarily require supportive and symptomatic care. Clinical management should focus on fever control, treatment of respiratory symptoms, serial assessment of hepatic, renal, and hematologic parameters, evaluation for coinfections, and careful documentation of exposure history, while preserving specimens for nucleic acid testing, serology, and viral whole-genome sequencing.

### 2.3 Case distribution, occupational exposure, and risk in agricultural populations

**Fig. 2:**
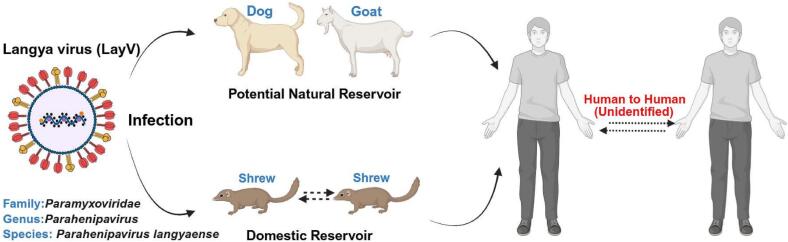
Potential transmission routes of LayV and their corresponding host associations.

LayV cases have mainly been reported from agricultural ecological regions, including Shandong and Henan, and farmers account for a substantial proportion of cases for whom occupational information is available [5]. This pattern is consistent with the basic logic of zoonotic spillover: field work, grain storage, activities in livestock enclosures, the movement of small mammals around households, and contact with environments contaminated by animal excreta may all increase opportunities for human exposure **(**Fig. 2**)**. Early public-health studies of LayV have likewise emphasized the need to systematically collect histories of animal contact and environmental exposure during surveillance for fever of unknown origin in rural and semi-rural regions.

Occupational exposure, however, is not equivalent to a transmission pathway. The high proportion of farmer cases may reflect genuine animal-contact risk, but it may also be influenced by sentinel-site placement, health-care-seeking behavior, regional industrial structure, and the sources from which samples were obtained. Future studies should adopt spatially and temporally integrated designs that place human cases, serological data from domestic animals, viral positivity rates in small mammals, seasonality, agricultural activities, and environmental samples within a unified analytic framework. Only when viruses from human cases are highly homologous to animal-derived viruses collected from the same geographic area and season, and when these findings align with well-defined exposure behaviors, can the spillover pathway of LayV be reconstructed with greater confidence.

### 2.4 Insufficient evidence for human-to-human transmission

The published literature to date has not confirmed sustained human-to-human transmission of LayV. The original cases lacked clear contact chains, and the limited data from household close contacts did not support stable transmission. LayV is therefore currently best understood as a cause of sporadic animal-to-human or environmentally mediated exposure events. By contrast, NiV outbreaks in some regions can be amplified through health-care caregiving, close contact, and postmortem handling; whether LayV is capable of low-frequency, context-dependent, or as-yet-undetected person-to-person transmission remains unknown [16], [17]. A useful public-health framework is that the risk posed by a major emerging pathogen typically depends on three capacities: the ability to infect humans, the ability to cause severe disease, and the ability to sustain human-to-human transmission. LayV has met the evidentiary threshold for human infection, but sufficient data are still lacking for the latter two capacities. Future studies should integrate close-contact follow-up, household-cluster investigations, seroconversion, viral-load dynamics, and symptom severity into prospective cohorts, rather than inferring transmissibility from scattered case reports.

### 2.5 Current limitations of epidemiological research

The epidemiological evidence for LayV remains subject to several major limitations. The number of recognized cases is small and geographically concentrated, making it difficult to estimate community infection rates, severe-disease rates, or case-fatality rates. Large-scale seroepidemiological data are lacking, leaving the proportions of previous infection and asymptomatic infection unknown. Standardized serological reagents and specific neutralization assays remain insufficient, and the risk of cross-reactivity needs to be controlled. Although animal-host data point toward shrews, the routes of viral shedding, the role of intermediate hosts, and the precise transmission pathways remain unresolved. Retrospective data and sentinel surveillance designs may also introduce selection bias. Given these limitations, LayV research should move beyond confirming the existence of the virus toward measuring its infection burden and transmission ecology. An optimal study design would include multicenter cohorts of fever of unknown origin, serological surveys in agricultural populations with high exposure risk, synchronized sampling of domestic animals and small mammals, and viral whole-genome sequencing for source tracing.

Existing evidence suggests that LayV cases show a degree of regional clustering, mainly in Shandong and Henan; however, this distribution may reflect either the true ecological niche of the virus or the effects of sentinel surveillance placement, sample provenance, and testing strategy [5]. Future work should not infer the geographic range of the virus solely from current case maps. Instead, methodologically harmonized cross-sectional and longitudinal surveillance should be conducted in shrews and other small mammals across the eastern coastal region, central China, and neighboring countries and regions. Negative results should also be incorporated into geographic risk models to reduce the influence of publication bias and detection bias on risk assessment.

## 3 Taxonomy, genome organization, and molecular evolution

### 3.1 Position of LayV within the family *Paramyxoviridae* and the genus *Parahenipavirus*

With continuing refinements in viral taxonomy, several non-bat-host-associated viruses that were previously discussed within the framework of henipaviruses or henipa-like viruses have now been incorporated into the conceptual scope of the genus *Parahenipavirus*. A recent review of the henipavirus host–virus interface noted that the ICTV has established *Parahenipavirus* as a new genus. Members of this genus are often associated with non-bat hosts, typically do not use ephrin receptors, and may contain additional open reading frames. LayV is classified as *Parahenipavirus langyaense* and represents one of the few viruses in this lineage for which human infection has been documented [18]. This taxonomic revision is not merely a change in nomenclature; it marks a shift in research paradigm. LayV should not be regarded simply as a less pathogenic variant of NiV or HeV, but rather as a member of a small-mammal-associated *parahenipavirus* lineage that has demonstrated the capacity to enter human populations.

### 3.2 Genome organization: the N–P–M–F–G–L coding framework and its functional significance

**Fig. 3:**
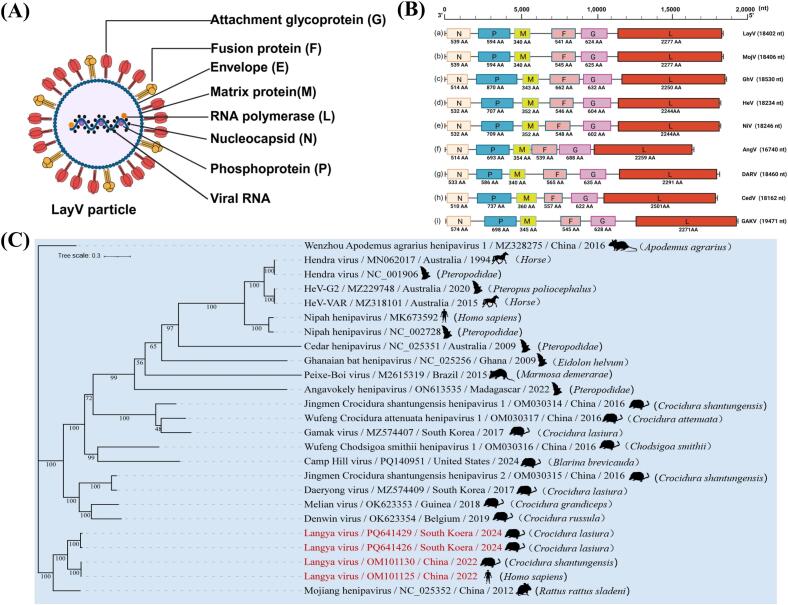
Virion architecture and genomic features of Langya virus (LayV). (A) Schematic representation of the LayV virion. (B) Comparison of genome organization among selected henipaviruses: (a) LayV, (b) MojV, (c) GhV, (d) HeV, (e) NiV, (f) AngV, (g) DARV, (h) CedV and (i) GAKV. Abbreviations: N, nucleocapsid protein; P, phosphoprotein; M, matrix protein; F, fusion protein; G, glycoprotein; L, large protein. (C) Phylogenetic tree of complete Langya virus genome sequences aligned with those of other henipaviruses. Complete viral genome sequences were collected and aligned using MAFFT v7.505. The phylogenetic tree was inferred using the maximum-likelihood method with 1000 bootstrap replicates on the IQ-TREE web server. GenBank accession numbers are shown for all reference sequences. Numbers on branches indicate bootstrap support values. The host species from which viral genomes were detected are also indicated.

LayV is an enveloped, nonsegmented, negative-sense RNA virus with a genome of approximately 18,402 bp. Its genome follows the canonical gene order of the family *Paramyxoviridae*, arranged from the 3′ to 5′ end as genes encoding the nucleocapsid protein (N), phosphoprotein (P), matrix protein (M), fusion protein (F), attachment glycoprotein (G), and large polymerase protein (L), separated by untranslated regions (UTRs). The N protein participates in nucleocapsid formation; the P/L protein complex mediates transcription and replication; the M protein contributes to viral assembly and budding; the F protein mediates membrane fusion and undergoes a conformational transition between prefusion and postfusion states, making it a major target for neutralizing antibodies and vaccine antigen design; and the G protein mediates host-cell recognition and fusion triggering, thereby shaping receptor usage, host range, and antigenic specificity. Unlike intracellular proteins that primarily influence replication and immune antagonism, the F and G proteins are directly exposed on the viral surface. They are therefore better suited as diagnostic antigens, vaccine immunogens, components for pseudovirus-entry assay construction, and targets for neutralizing-antibody screening. In addition, on the basis of shared henipavirus mechanisms and LayV genome annotation, the P gene may generate nonstructural proteins such as C, V, and W through RNA editing and related coding strategies; however, the immune-antagonistic functions of these proteins in LayV remain to be experimentally validated [18] **(**Fig. 3A**)**.

### 3.3 Phylogenetic relationships with Mojiang virus, Gamak virus, and Daeryong virus

Comparative analysis of LayV with small-mammal-associated viruses such as Mojiang virus (MojV), Gamak virus (GAKV), and Daeryong virus (DARV) is central to defining its evolutionary position. MojV was first identified in rodent samples from Mojiang, Yunnan Province, China, expanding the known host range of henipa-like viruses and indicating that non-bat small mammals can harbor viruses related to highly pathogenic henipaviruses [19]. Subsequent reviews of shrew-associated henipa-like viruses and surveillance studies from South Korea further indicate that shrew-derived parahenipaviruses are not isolated anomalies, but may instead represent an important branch of the small-mammal virome in East Asia [20], [21], [22].

The distinctive importance of LayV lies in its linkage of this animal-virus lineage to human febrile disease. Many parahenipaviruses are currently supported only by animal sequence data and lack viral isolation, serological evidence, or infection-model validation. LayV, by contrast, provides a rare clue to human infection. It can therefore serve as a critical reference point for comparing the cross-species potential of MojV, GAKV, DARV, and other parahenipaviruses **(**Fig. 3B and C**)**.

### 3.4 Significance of genomic surveillance for detecting cryptic spillover

The discovery of LayV demonstrates that broad-spectrum RT-PCR, metagenomic sequencing, and targeted amplicon sequencing can identify previously unknown viruses in cases of fever of unknown origin and in animal specimens that test negative by routine diagnostics. Early broad-spectrum paramyxovirus RT-PCR assays provided an important technical foundation for screening unknown paramyxoviruses [23]. Future genomic surveillance should not stop at the discovery of new sequences. Instead, it should be integrated with functional assays, pseudovirus-entry studies, receptor screening, and assessment of serological cross-reactivity, thereby transforming sequence discovery into a tool for risk stratification.

## 4 Animal hosts, ecological niches, and mechanisms of cross-species spillover

### 4.1 Evidence supporting shrews as candidate natural hosts

**Fig. 4:**
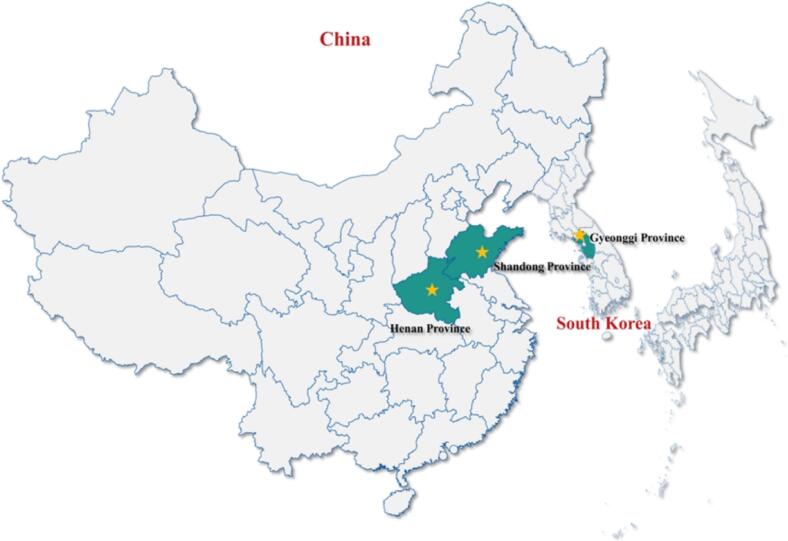
Geographic distribution of LayV emergence and zoonotic detection.

The original study screened 25 species of wild small animals for LayV RNA and detected viral RNA in three rodent species and two shrew species, suggesting that shrews currently represent the strongest clue to the natural host. Among 262 tested shrews, approximately 27% were positive for LayV RNA. Low-level seropositivity was also detected in dogs and goats, indicating that these animals may participate in the ecological cycle of LayV or at least serve as sentinels of exposure [8]. Regional viromic studies further support this interpretation. A recent metagenomic sequencing study of lung tissue from wild shrews collected across six provinces and municipalities along the eastern coast of China identified 54 known viruses and 72 novel viruses, including LayV and other viruses with potential spillover risk. This study suggests that shrews harbor highly diverse RNA-virus communities and represent a small-mammal viral reservoir that should not be overlooked at agricultural ecological interfaces in East Asia [24]. Investigators in South Korea retrospectively analyzed metagenomic sequencing data from kidney samples of two Ussuri white-toothed shrews (*Crocidura lasiura*) collected in 2017 and subsequently screened kidney samples from the same shrew species collected in 2023 for paramyxoviruses. They detected LayV in 12.5% of samples (3/24) [21] **(**Fig. 4**)**. By contrast, investigators in Belgium did not detect LayV genomic sequences by third-generation sequencing of 17 local shrew samples, suggesting that the spatial distribution of LayV or closely related viruses may vary by region [25]. Together, these findings indicate that LayV may not be confined to the Chinese regions in which human cases have been reported, but its distribution across East Asia and beyond is likely to be heterogeneous.

It is important to emphasize that detecting viral RNA is not equivalent to confirming a natural host. Establishing natural-host status requires evidence of stable infection, a relatively high field-detection rate, viral replication or isolation, host seropositivity, defined shedding sites, and spatiotemporal linkage with the exposure locations of human cases. Current evidence supports shrews as the strongest candidate hosts for LayV, but remains insufficient to reconstruct the complete transmission chain. Negative results from small-scale shrew sequencing in some regions are also informative: they may indicate regional restriction in LayV distribution, or they may reflect differences in sample size, host species, sampled organs, and sequencing depth. Future work should therefore avoid defining LayV simplistically as either “globally distributed” or “restricted to Shandong and Henan.” Instead, standardized sampling and viromic methods should be used to construct regional risk maps.

### 4.2 From the classical fruit bat–amplifying host paradigm to a shrew–amplifying host framework

Traditional henipavirus risk assessment has long been built around the classical “fruit bat–amplifying host–human” transmission paradigm. In the ecology of NiV and HeV, fruit bats serve as natural reservoirs, whereas domestic animals such as pigs and horses can act as important amplifying hosts for human infection. NiV can also spread further through bat-contaminated food, close contact, and health-care-associated caregiving, making large domestic animals and high-risk exposure settings central to outbreak warning and transmission interruption [16], [26].

The discovery of LayV and related parahenipaviruses, however, indicates that not all henipa-like viral ecological risks can be interpreted through a bat-centered framework. Studies of genetic diversity and geographic dispersal show that the host range and distribution of henipavirus-related viruses are continuing to expand. Beyond fruit bats, shrews, rodents, and other small mammals may also constitute important viral reservoirs [27]. In contrast to NiV and HeV, LayV has not yet been shown to involve fruit bats, nor has a strong amplifying host been identified. Its reported cases are more consistent with sporadic zoonotic or environmental exposure events. The association of cases with agricultural populations further suggests that the spillover interface may lie within agricultural ecosystems where field work, grain storage, livestock enclosures, residential environments, and small-mammal habitats intersect.

Cross-species transmission is not determined by a single host species. Instead, it is shaped by host density, contact frequency, viral shedding level, environmental stability, land-use change, shifts in species distribution, receptor compatibility, and other interacting factors [28], [29]. Within this framework, seropositive signals in dogs and goats raise a question that warrants systematic follow-up: are these domestic animals intermediate hosts or amplifying hosts in the LayV transmission chain, or do they merely reflect incidental infection after environmental exposure or serological cross-reactivity? Current evidence is insufficient to establish dogs or goats as amplifying hosts. These seropositive findings require further validation through animal neutralization assays, nucleic acid testing, exposure-history investigation, longitudinal serological monitoring, and experimental infection studies.

Accordingly, LayV control strategies cannot simply replicate the NiV/HeV model. Surveillance must expand from “fruit-bat-associated classical henipaviruses” to “small-mammal-associated parahenipaviruses.” Within a One Health framework, surveillance should simultaneously include Crocidura shrews and other small mammals, domestic animals such as dogs and goats, environmental samples from farmland and granaries, agricultural activities, geoclimatic variables, and human populations with fever of unknown origin or respiratory symptoms after animal exposure. Only by integrating viral genomics, serology, spatial geography, seasonality, host density, and human behavioral data can we determine whether domestic animals are transmission-chain nodes, exposure sentinels, or incidental bystanders at the ecological interface. Such integration would ultimately establish a closed-loop system linking animal viral circulation, agricultural environmental exposure, sporadic human infection, sentinel surveillance detection, and reverse tracing of animal sources [26], [30] **(**Fig. 1B**)**.

## 5 Viral entry mechanisms: noncanonical receptor usage and structural-biology breakthroughs

### 5.1 From ephrin-dependent entry to recognition of an unknown receptor

Paramyxovirus entry is typically mediated by coordinated action between an attachment glycoprotein and a fusion protein. In prototypic NiV and HeV, the G glycoprotein recognizes ephrin-B2 or ephrin-B3, initiating conformational signaling that triggers the F protein to transition from a metastable prefusion state to a postfusion conformation, thereby driving fusion between the viral envelope and the host-cell membrane [31], [32], [33]. This “ephrin-receptor recognition–G/F-coupled triggering–membrane fusion” framework explains the tissue tropism of NiV and HeV and has guided the design of neutralizing-antibody strategies. However, it cannot be directly extrapolated to LayV.

Together with MojV and other atypical henipa-like viruses, LayV suggests that parahenipaviruses may use receptor-engagement and fusion-triggering pathways distinct from those of classical henipaviruses. The MojV attachment glycoprotein can mediate a host-cell entry route that differs from that used by NiV and HeV [PMID:28699636], and LayV has subsequently been shown not to depend on canonical ephrin receptors. The central breakthrough in LayV-G biology is the demonstration that it does not bind ephrin-B2 or ephrin-B3. A 2024 study in *Structure* resolved the 2.77 Å crystal structure of the C-terminal domain of LayV G, showing that it more closely resembles MojV-G than NiV-, HeV-, or CedV-G. Surface plasmon resonance assays and structural docking analyses both indicated that the LayV-G CTD does not bind ephrin-B proteins, with steric hindrance and differences in surface electrostatic potential likely impeding ephrin-B engagement [34].

This finding is reinforced by cryo-EM analysis of the LayV-G tetramer: although LayV-G retains the overall assembly architecture of an attachment glycoprotein, it does not use the canonical ephrin-B2/B3 receptors, indicating that cross-species entry may rely on an unknown receptor or a distinct host-recognition interface [35]. The significance of LayV-G structural biology therefore extends beyond providing a template for antigen design. More fundamentally, it establishes “unknown receptor recognition” as a central question in LayV infection biology, tissue tropism, host-range prediction, and spillover-risk assessment.

### 5.2 LayV-F conformational transition, fusion-peptide release, and membrane-fusion triggering

**Fig. 5:**
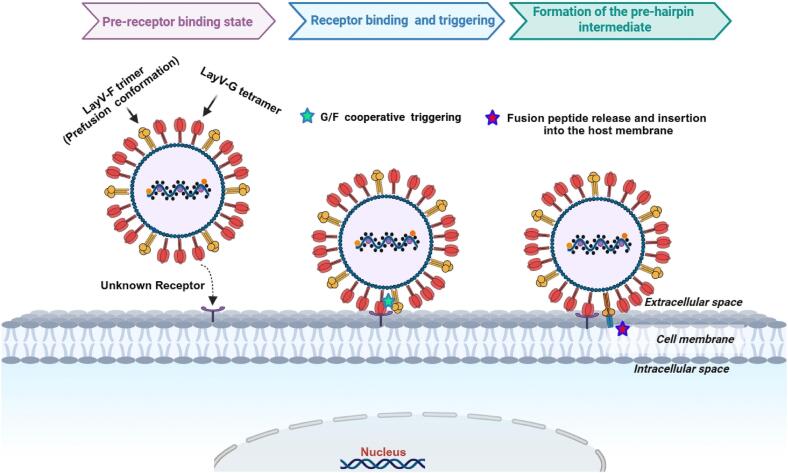
Proposed model of LayV-G-mediated triggering of LayV-F conformational rearrangement during viral entry. Upon engagement with an unknown receptor, LayV-G (red) triggers the transition of LayV-F from the prefusion conformation to a prehairpin intermediate; the triggering interaction is marked by a blue asterisk. The LayV G–F interaction induces conformational rearrangement of LayV-F, allowing the fusion peptide to adopt a “spring-loaded” state and form a hairpin-like structure that inserts into the host-cell membrane, as indicated by the purple asterisk. This process ultimately drives membrane fusion and viral entry. (For interpretation of the references to colour in this figure legend, the reader is referred to the web version of this article.)

Structural studies of LayV-F connect noncanonical receptor recognition to the mechanics of membrane fusion. A 2023 study in Journal of Virology resolved cryo-EM structures of the uncleaved LayV-F ectodomain in both prefusion and postfusion conformations. The results showed that LayV-F broadly follows the conserved conformational-transition mechanism characteristic of paramyxovirus F proteins, but that the surface properties at the apex of the prefusion trimer differ, potentially influencing antigen accessibility. In the prefusion state, the fusion peptide is buried within a highly conserved hydrophobic interprotomer pocket and adopts a “spring-loaded” configuration, suggesting that the prefusion-to-postfusion transition may require perturbation of this pocket and release of the fusion peptide [36] **(**Fig. 5**)**.

These structural data should be interpreted as mechanistic evidence, not as direct evidence of vaccine efficacy. LayV-F demonstrates that parahenipaviruses retain the shared paramyxovirus fusion machinery, yet local differences in surface architecture may alter antibody accessibility and the threshold for fusion triggering. Key unresolved questions include how binding of the unknown receptor to G is transmitted to F; whether F processing, trafficking, and triggering efficiency constrain LayV replication across species and tissues; and whether F/G interactions determine LayV cell tropism and cross-species entry capacity.

### 5.3 Mechanistic gaps: the unknown receptor, tissue tropism, and tiered experimental models

The most important gap in LayV entry research remains the identity of its receptor. This unknown limits inference about tissue tropism, animal-model selection, and transmission risk. Reviews of henipavirus tissue tropism indicate that differences in viral tropism across species, organs, and cell types are closely linked to receptor distribution, immune evasion, and viral replication efficiency [37]. Reviews of F-protein trafficking and activation further suggest that henipaviruses, parahenipaviruses, and henipa-like viruses differ in F-protein processing, transport, and triggering, and that these differences may influence cell–cell fusion efficiency and host adaptation [38].

Future studies should therefore prioritize identification of the LayV receptor through CRISPR knockout screening, expression cloning, affinity-purification mass spectrometry, and cross-species cellular-susceptibility analyses. In parallel, tiered pseudovirus, virus-like particle, and infectious-virus models should be established to evaluate F/G-mediated entry, antibody neutralization, and cellular and tissue tropism under biosafety-appropriate conditions. Only by integrating receptor identification, structural analysis, and functional validation can the field move beyond the conclusion that LayV entry is “non-ephrin-dependent” and toward a mechanistic model capable of predicting host range and transmission risk.

## 6 Diagnostic, vaccine, and antibody countermeasures

### 6.1 Molecular and serological diagnostics

The discovery of LayV illustrates the complementary roles of targeted and untargeted molecular diagnostics in the identification of emerging pathogens. Targeted RT-PCR remains the preferred method for acute detection, whereas metagenomic sequencing and broad-spectrum paramyxovirus RT-PCR are most useful for pathogen discovery and assay development [23]. A quadruplex RT-qPCR assay for LayV, MojV, NiV, and CedV has shown favorable analytical sensitivity, specificity, and reproducibility [39].

A practical three-tiered framework would combine RT-PCR or isothermal amplification for acute case finding **(**Fig. 1C**)**, whole-genome sequencing for source tracing and variant surveillance, and serology/neutralization assays for prior exposure. Preliminary indirect ELISAs based on NiV and LayV proteins can detect antibodies in animals, but species-specific cutoffs, cross-reactivity, and concordance with neutralization assays require further validation [40]. Standardized positive controls, reference sera, and multicenter performance datasets remain essential.

### 6.2 CRISPR-Cas12a and RPA-based on-site detection platforms

Rapid assays may extend LayV testing beyond conventional laboratories. An RPA-Cas12a workflow detected 10 copies/μL within 30 min at room temperature, whereas an HRP-ssDNA reporter enabled amplification-free visual detection at 1200 copies/μL [41]. Separately, an N-gene RPA-lateral-flow assay achieved a reported detection limit of 1.22 copies/μL after 30 min at 42 °C and showed no cross-reactivity with the tested HeV, NiV, or Sendai virus controls [42]. These proof-of-concept platforms are promising for low-resource clinical testing and animal surveillance, but analytical performance on contrived templates and testing of limited viral RNA of infected cells do not establish diagnostic accuracy in humans. Multicenter evaluation should therefore use prospectively collected clinical, animal, and environmental specimens and assess sample preparation, contamination control, stability, operator reproducibility, and agreement with reference RT-qPCR.

### 6.3 Structure-guided vaccine antigen development

Two in silico studies proposed multi-epitope LayV vaccine constructs [43], [44]. These analyses can prioritize B- and T-cell epitopes, but they do not demonstrate antigen expression, immunogenicity, neutralization, or protection. Experimental development should instead proceed from the structurally defined F and G glycoproteins. As previous studies reported, LayV F/G are antigenically distinct from NiV/HeV, and antibodies elicited by prototypic HNV glycoproteins show limited recognition of LayV [45], [46]. The most defensible antigen-engineering strategies are prefusion stabilization of F, conformational presentation of the G head or tetramer, and comparative evaluation of combined F/G immunogens. Engineered disulfide bonds can stabilize LayV F in the prefusion state, supporting the technical feasibility of this approach [34], [35], [36], [46], [47].

NiV and HeV programs provide platform-level experience with G-protein subunits, viral vectors, mRNA vaccines, and clinical translation, but they cannot be treated as evidence of LayV protection [48], [49], [50]. A staged program should first establish LayV-specific pseudovirus neutralization assays and animal immunization models, then compare conserved epitopes across LayV, MojV, GAKV, DARV, and prototypic HNVs before advancing broader pan-parahenipavirus or pan-henipavirus candidates.

### 6.4 LayV-specific monoclonal antibody strategies

Studies of prototypic NiV and HeV have established the feasibility of G-directed passive immunization. The m102.4 antibody and other G-specific antibodies have shown potent neutralization, protection in animal models, and, for m102.4, phase I safety evaluation [51], [52], [53], [54], [55]. However, marked antigenic divergence means that these antibodies should not be presumed to neutralize LayV. Recent LayV-G studies identified specific conformational epitopes and noncompeting monoclonal antibodies that bind distinct head-domain surfaces and exhibit Fc-mediated effector activity, providing a direct basis for LayV-specific antibody design [56], [57]. Priorities include receptor-blocking antibodies, combinations that occupy complementary G surfaces or disrupt G-F triggering, and evaluation of neutralization and Fc functions in pseudovirus, authentic-virus, and animal models. Until the cellular receptor is identified, mechanistic claims of receptor blockade should remain provisional.

## 7 Discussion and future perspectives

Against the backdrop of global climate change, ecological transformation, and the continuing expansion of human activities, the risk of viral cross-species transmission is increasing, and the emergence of infectious diseases is being shaped by increasingly complex ecological drivers [58]. As a newly identified zoonotic *parahenipavirus*, LayV is now recognized as a potential threat to human health. Research on LayV is moving from pathogen discovery toward mechanistic elucidation and the construction of countermeasure platforms. Compared with NiV and HeV, LayV has not caused outbreaks with high case-fatality rates, nor has sustained human-to-human transmission been confirmed. Its short-term public health risk should therefore not be overstated. Yet it would be equally misleading to disregard its scientific value and early-warning significance solely because the number of recognized cases remains small. The true importance of LayV lies in its integration of evidence for human infection, shrew-host associations, non-ephrin receptor-mediated entry, and structure-guided countermeasure strategies within a single conceptual framework. In doing so, LayV shifts parahenipaviruses from a peripheral branch of animal viromics to a new model for understanding cross-species transmission among emerging paramyxoviruses. Integrating clinical recognition, laboratory diagnostics, and primary-level public health strategies will help move LayV research beyond “spillover ecology and structural mechanisms” toward a closed-loop framework encompassing ecological discovery, clinical identification, laboratory testing, structure-guided countermeasure development, and field surveillance [59]. This integrated approach preserves the conceptual novelty of LayV as a model of *parahenipavirus* spillover while enhancing the practical relevance of the Review for clinical practice and public health deployment.

From a One Health perspective, LayV underscores the need to extend emerging-pathogen surveillance deeper into agricultural ecosystems and small-mammal interfaces. Traditional henipavirus risk assessment has often centered on fruit bats, pigs, horses, and hospital-associated transmission chains. This paradigm is appropriate for NiV and HeV, but it is insufficient to capture the shrew-associated *parahenipavirus* ecology represented by LayV. Future surveillance should integrate human febrile cohorts, shrew viromes, livestock serology, environmental samples, and geoclimatic variables within a unified study design to reconstruct the pathway by which viruses move from wildlife reservoirs into human populations. Community-level prevention and control should be guided by risk stratification and exposure reduction. In regions where LayV cases have been previously identified or where shrew-positive signals are strong, LayV can be incorporated into investigations of fever of unknown origin and animal-exposure history. Health education should be directed toward farmers, field workers, and personnel managing livestock enclosures and granaries. Gloves, masks, and environmental disinfection should be emphasized during rodent control, granary cleaning, or the handling of small-mammal carcasses. At the same time, crude interventions aimed at “eliminating hosts” should be avoided. Instead, environmental management, risk communication, and integrated animal–human surveillance should be used to reduce opportunities for contact.

From the perspective of countermeasure translation, LayV research should avoid two extremes: completely postponing tool development because case numbers are low, and prematurely advancing product development in the absence of models and defined clinical need. The translation of LayV countermeasures faces several bottlenecks. Standardized serum panels are lacking, limiting the evaluation of diagnostic specificity and cross-reactivity. Unified antigen standards have not been established, and the immunological performance of F, G, the G head domain, G tetramers, and prefusion F has not been systematically compared. Infectious animal models remain insufficient, preventing rigorous evaluation of vaccine- and antibody-mediated protection. Protective immune correlates have not been defined. Biosafety constraints and limited sample access further restrict multicenter evaluation. Reviews of henipavirus countermeasures have emphasized that outbreaks caused by these pathogens are sporadic and unpredictable, making conventional phase III clinical-trial pathways potentially impractical. Future development may require conditional approval, emergency-use authorization, or animal-rule pathways, together with global, regional, and endemic-country stockpiles supported by equitable pricing mechanisms [1]. This perspective is especially relevant to LayV. Its case numbers are even smaller and its transmission chains less clearly defined, making it unlikely that product efficacy can be evaluated in the near term through conventional clinical endpoints. Priority should therefore be given to establishing standardized RT-PCR and field-deployable nucleic acid assays, structure-guided F/G antigens, pseudovirus neutralization assays, monoclonal-antibody screening platforms, receptor-identification systems, animal models, and immune-correlate frameworks. Even if LayV itself does not evolve into a large-scale threat, these platforms could be rapidly adapted to MojV, GAKV, DARV, and newly discovered parahenipaviruses.

Future LayV research should prioritize six tasks. First, standardized case definitions and multicenter seroepidemiological studies should be established to define the true burden of infection, the proportion of asymptomatic infection, and the full spectrum of clinical severity. Second, systematic animal surveillance should be conducted across East Asia and other regions where shrews are distributed, with the aim of confirming natural hosts, shedding sites, seasonality, and spatial distribution, tracing viral origins and evolutionary trajectories, and identifying key genetic variants to provide a scientific basis for outbreak early warning. Third, the LayV receptor should be identified as rapidly as possible, and the structure of the receptor–G-protein complex should be resolved to clarify host range, tissue tropism, and the mechanisms of cross-species entry. Fourth, candidate therapeutic strategies for LayV infection should be developed. On the basis of mechanisms governing viral replication, entry, and immune evasion, high-throughput drug screening and AI-assisted drug design should be combined to identify small molecules, peptides, antibodies, and other candidates with specific antiviral activity, followed by pharmacodynamic and toxicological evaluation [60] **(**Fig. 1C**)**. Fifth, structure-based vaccine development should be accelerated by optimizing antigen selection, conformational stabilization, and delivery platforms. Particular attention should be given to evaluating the immunogenicity, safety, and cross-protective potential of prefusion F, the G head domain, G tetramers, and combined F/G antigens, with a stepwise path toward subunit, viral-vector, and mRNA vaccines with broad anti-parahenipavirus or pan-henipavirus potential [61], [62], [63], [64]. Sixth, experimental animals susceptible to LayV should be identified, and gene-editing technologies should be used to construct animal models that more closely recapitulate human infection. Such models will be essential for studying viral replication, transmission, tissue tropism, and pathogenesis in vivo, while also enabling evaluation of the protective efficacy of drugs, vaccines, and antibodies and providing a foundation for subsequent clinical research [65].

In summary, the discovery of LayV has created new questions and opportunities for virology and public health preparedness. As a newly identified zoonotic *parahenipavirus*, LayV substantially expands our understanding of host range, receptor usage, and spillover ecology within the family *Paramyxoviridae*. It reminds us that emerging viral risk does not arise only from the re-emergence of known pathogens with high case-fatality rates, but may also originate from long-overlooked small-mammal viromes, low-frequency sporadic infections, and noncanonical mechanisms of viral entry. Positioning LayV at the intersection of *parahenipavirus* spillover ecology, non-ephrin receptor-mediated entry, and structure-guided countermeasure development will help build a more anticipatory warning and prevention system for emerging paramyxoviruses.

## Generative AI statement

The author(s) declared that generative AI was not used in the creation of this manuscript.

## CRediT authorship contribution statement

**Liang Shen:** Writing – original draft. **Yanfei Tong:** Writing – original draft. **Wenqi Chai:** Writing – original draft. **Jiali Sun:** Writing – original draft. **Jianzhong Zhao:** Writing – original draft. **Lijuan Yin:** Writing – original draft. **Yang Yang:** Writing – review & editing. **Chunhua Wang:** Writing – review & editing. **Wenjie Tan:** Writing – review & editing. **Ji Zhang:** Writing – review & editing.

## Funding

The author(s) declared that financial support was received for this work and/or its publication. This research was supported by 10.13039/501100001809National Natural Science Foundation of China (82002192), 10.13039/501100003819Projects of Natural Science Foundation of Hubei Province (2025AFD033, 2025AFD045), Science and technology research program of Education Department of Hubei Province (Q20242107), Talent Development Program of Xiangyang Central Hospital (2025RCLJ-011, 2025RCQM-078).

## Declaration of competing interest

The authors declare that they have no known competing financial interests or personal relationships that could have appeared to influence the work reported in this paper.

## Data Availability

No data was used for the research described in the article.
